# Supplementary material for: Single molecule demonstration of Debye–Stokes–Einstein breakdown in polystyrene near the glass transition temperature
Source: Nat Commun. 2022 Jun 23;13:3580. doi: 10.1038/s41467-022-31318-z (PMC9226357; doi:10.1038/s41467-022-31318-z)
Supplement: Supplementary file 1 — Supplementary Information [file 41467_2022_31318_MOESM1_ESM.pdf]

Supplementary Information

Single Molecule Demonstration of Debye-Stokes-Einstein Breakdown in Polystyrene near the Glass Transition Temperature

Nicole L. Mandel, Soohyun Lee, Kimyung Kim, Keewook Paeng and Laura J. Kaufman

Supplementary 1 - Rotational Dynamics as Obtained from Single Molecule Measurements	2
Supplementary Figure 1	
Supplementary 2 - Estimation of Localization Error	3
Supplementary Note	
Supplementary Figure 2	
Supplementary 3 - Translational Dynamics as Obtained from Single Molecule Measurements	5
Supplementary Note	
Supplementary Figure 3	
Supplementary 4 - Quasi-Ensemble Assessments of Rotation and Translation	7
Supplementary Figure 4	
Supplementary 5 - Imaging Fluorescence Correlation Microscopy Data	8
Supplementary Figure 5	
Supplementary 6 - Additional Debye-Stokes-Einstein Breakdown Information	9
Supplementary Figure 6	
Supplementary 7 - Debye-Stokes-Einstein Behavior and Assumptions	10
Supplementary Note	
Supplementary Figure7	
Supplementary 8 - Additional Single Molecule Scatter Plots	11
Supplementary Note	
Supplementary Figure 8	
Supplementary 9 - Translational Diffusion Coefficients of Molecules Stratified by Mobility	13
Supplementary Note	
Supplementary Figure 9	
Supplementary 10 - Additional Characteristics of Mobile and Immobile Molecules	14
Supplementary Figure 10	
Supplementary 11 - Step Size Distributions	15
Supplementary Figure 11	
Supplementary References	16

Supplementary 1 - Rotational Dynamics as Obtained from Single Molecule Measurements

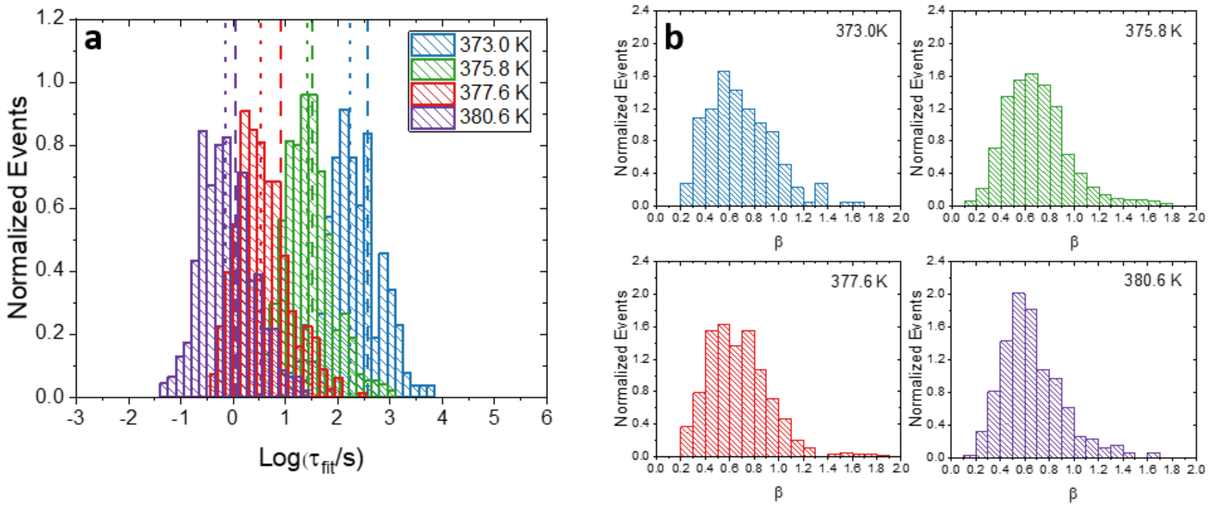

Supplementary Figure 1. (a) Rotation time, τ_{fit}, and (b) stretching exponent, β, distributions for single molecule rotation at the four temperatures studied. In (a) dashed vertical lines are median values. Median β values ranged between 0.61-0.66, with no clear trend as a function of temperature, a finding consistent with previous measurements on pPDI in 168 kg/mol polystyrene at these trajectory lengths and over similarly narrow temperature ranges.[1,2]

Supplementary 2

Supplementary Note: Estimation of Localization Error

Localization error was estimated both theoretically and empirically. First, following Mortensen et. al, Equation 1 was used to quantify static localization error, σ.[3,4]

$$\sigma^2 = \frac{\sigma_a^2}{N}\left[\frac{16}{9} + \frac{8\pi\sigma_a^2 b^2}{q^2 N}\right] \qquad (1)$$

Here, $\sigma_a^2 = s^2 + \frac{q^2}{12}$ and $s = 0.55\lambda_{em}/2(1.17 \cdot NA)$, where s is the standard deviation of the point spread function (PSF), q is pixel size in nanometers, λ_{em} is emission wavelength of the probe in nanometers, NA is numerical aperture of the objective lens, N is the number of photons collected, and b is the background noise per pixel. Using q = 169 nm, NA = 0.95, λ_{em} = 556 nm, and N and b estimated by backwards conversion of pixel intensity counts to photons,[5] localization error was calculated to be ≈ 11 nm for 2 s exposure time. Following Michalet, dynamic localization uncertainty may be estimated from the static localization uncertainty by, $\sigma_{dynamic} = \sigma\sqrt{1 + \frac{D_T t_\varepsilon}{s^2}}$, where σ is the static localization error, s is as defined above, D_T is the diffusion coefficient and t_ε is exposure time.[6] Using the median diffusion coefficient obtained at the highest temperature studied via single molecule experiments, 380.6 K with 1 s exposure, the dynamic localization error is ≈ 13 nm. Given that this is the upper limit of error due to probe dynamical motion, we conclude that static localization error dominates localization error for the conditions used in this study.

Experimentally, movies of N,N'-dipentyl-3,4,9,10-perylenedicarboximide (pPDI) probes in 168 kg/mol, polystyrene were taken at 300.0 K (T_g – 74.3 K) for 2000 s at 0.5 Hz continuously to estimate localization error and related quantities. Localization error was quantified following tracking of individual molecules, with typical trajectories being 70 frames. Median step size obtained from these trajectories was found to be 9 nm. This finding was independent of time between frames (TBF), with native time between frames of 2 s and longer time between frames achieved by removing frames from the movie before identifying molecules and linking their positions into trajectories (**Supplementary Fig. 2a**). These results are in good agreement with the theoretical prediction.

Despite step size distributions that remain stable as a function of TBF, this experimental variable does affect apparent width of the diffusion coefficient distribution when diffusion coefficients are obtained from single molecule mean square displacements (**Supplementary Fig. 2b**). Additional discussion of dependence on time between frames is included in **Supplementary 3**. Radius of gyration (R_g) calculated as described in the **Methods** section of the main text for individual trajectories associated with the 2 s TBF trajectories from this data set are shown in **Supplementary Fig. 2c**, with a median value of 2.2 nm.[7]

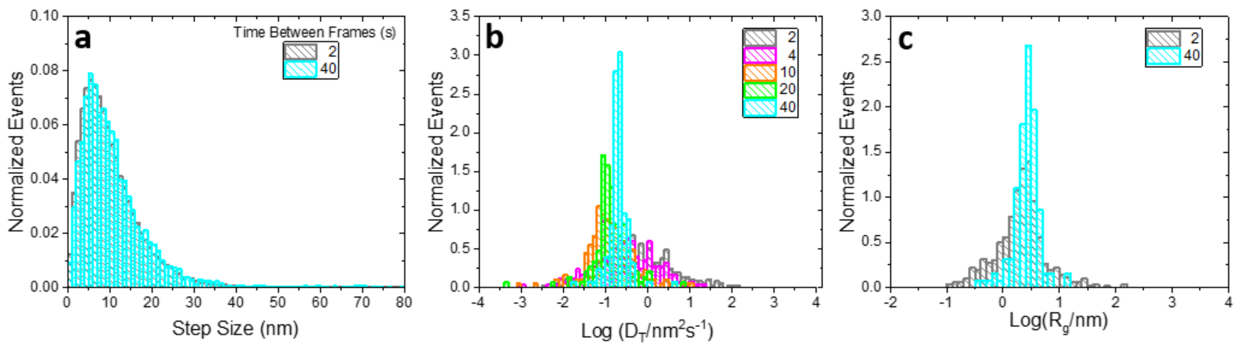

Supplementary Fig. 2. (a) Step size distributions for molecules tracked at 300.0 K at 2 s and 40 s time between frames (TBF). Median and distribution width are invariant to TBF. (b) Diffusion coefficient (D_T) distributions for molecules tracked at 300.0 K at 5 different TBF. As a function of increasing TBF, distribution width decreases. (c) Distributions of radii of gyration (R_g) for molecules tracked at 300.0 K at 2 s and 40 s TBF. Median is invariant to TBF while distribution width decreases with increasing TBF.

Supplementary 3

Supplementary Note: Translational Dynamics as Obtained from Single Molecule Measurements

As for data collected far below T_g (**Supplementary 2**), data collected at temperatures above T_g was analyzed as a function of time between frames (TBF) to assess possible effects of this parameter on distribution width and median diffusion coefficient. At each temperature studied, multiple TBFs were analyzed for the same movies by deleting intermediate frames. While the distribution width of D_T values changed substantially as a function of TBF for molecules detected at 300.0 K (**Supplementary Fig. 2b**), at higher temperatures this was not the case (**Supplementary Fig. 3a**). At 300.0 K, as molecules may be assumed to be immobile, any difference in D_T distribution width with increasing TBF is assumed to be due to noise and the ways in which noise influences D_T calculations. Although a similar level of noise is present in movies collected below and above T_g, decrease in distribution width is seen only at 300.0 K, suggesting the wide D_T distributions seen at higher temperatures are not determined by noise, particularly when movies are analyzed at relatively large TBF.

While the width of the diffusion coefficient distribution decreases for molecules measured at 300.0 K with increasing TBF, the median value of the diffusion coefficient itself is stable, reflecting the noise floor of the measurement, as described in **Supplementary 2**. In contrast, at higher temperatures median diffusion coefficient obtained decreases as TBF increases, consistently following a dependence characterized by $D_T = 74.8(\mathrm{TBF})^{-1.14}$ (**gray trendline, Supplementary Fig. 3b**).

Noting that such behavior could emerge from a variety of sources, we further assessed median diffusion coefficient in a variety of manners. First, in an attempt to suppress possible noise contributions to obtained diffusion coefficients, instead of removing frames to achieve movies with greater TBF, frames between assessed points were summed before feature finding and particle tracking. Calculations associated with equations presented in **Supplementary 2** suggested this would improve static localization accuracy while having minimal impact on dynamic localization accuracy. Assessing diffusion coefficients in this manner at select TBF values did not significantly change median diffusion coefficients obtained (**Supplementary Fig. 3b, stars**).

Additionally, we conducted a frame rate sweep, in analogy to that described for rotational measurements in Reference 8 to determine a single median diffusion coefficient using data from movies at all TBFs assessed. This was informed by the recognition that the same molecules were not necessarily assessed at each TBF even though the raw data was identical, potentially leading to sub-ensemble selection, with faster molecules being overrepresented in movies with shorter TBF and slower molecules overrepresented in movies with longer TBF. Moreover, in each movie, molecules could be found (and lost) more than once in the particle tracking process. To eliminate both issues, a frame rate sweep analysis was performed that assured one instance of each molecule found was preserved across all TBF. In this analysis, for each molecule (identified by its position) within a movie or across movies that differed only in TBF analyzed, the instance chosen was that which yielded the lowest diffusion coefficient. This instance was chosen as it should provide a lower bound on translational enhancement over rotation at each temperature.

This method resulted in distributions with significant contributions from every TBF studied at each temperature, yet both the distribution of and the median D_T values at each temperature remained similar to that found at TBF = τ_{fit}. (**Supplementary Fig. 3b, open circles**). Given these findings, data taken at TBF = τ_{fit} is used for further analysis except where noted, as this yields diffusion coefficients similar to those of other approaches investigated and provides constant average rotational motion between assessed positions across temperatures.

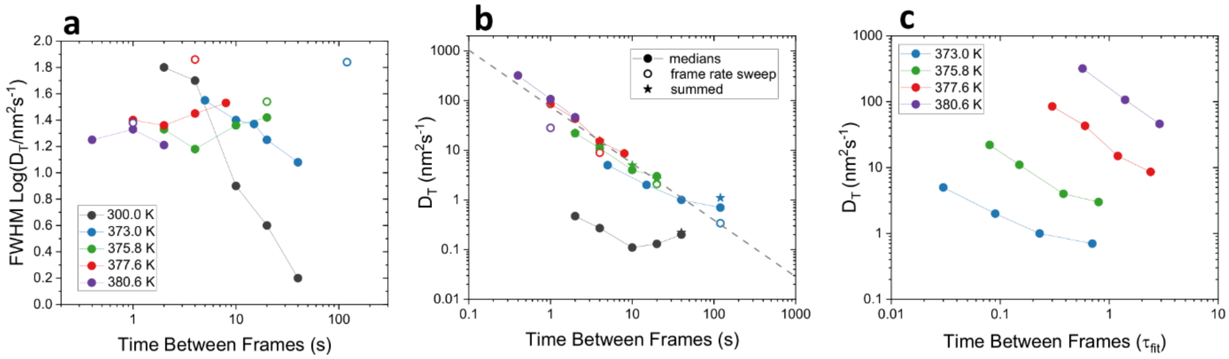

Supplementary Fig. 3. (a) Full width at half max (FWHM) of diffusion coefficient distributions at each temperature studied as a function of TBF. Data collected at 300.0 K shows a clear decrease in FWHM as a function of TBF, an effect not seen at the four other temperatures. Open circles indicate FWHMs obtained from frame rate sweep distributions, which tend to be wider than single-TBF distributions. (b) (filled circles) Median diffusion coefficients D_T as a function of TBF. Median D_T decreases with TBF for all temperatures except 300.0 K. (stars) Median D_T values obtained when intermediate frames are summed rather than deleted to increase TBF. (open circles) Median D_T values from frame rate sweep analysis at each temperature. Each point contains molecules assessed at each TBF but are placed on the x axis to correspond with TBF = τ_{fit} for comparison. Gray dashed line is the best fit of log(D_T) vs. log(TBF) data. (c) Median diffusion coefficient D_T as a function of TBF in terms of τ_{fit}.

Supplemenary 4 - Quasi-Ensemble Assessments of Rotation and Translation

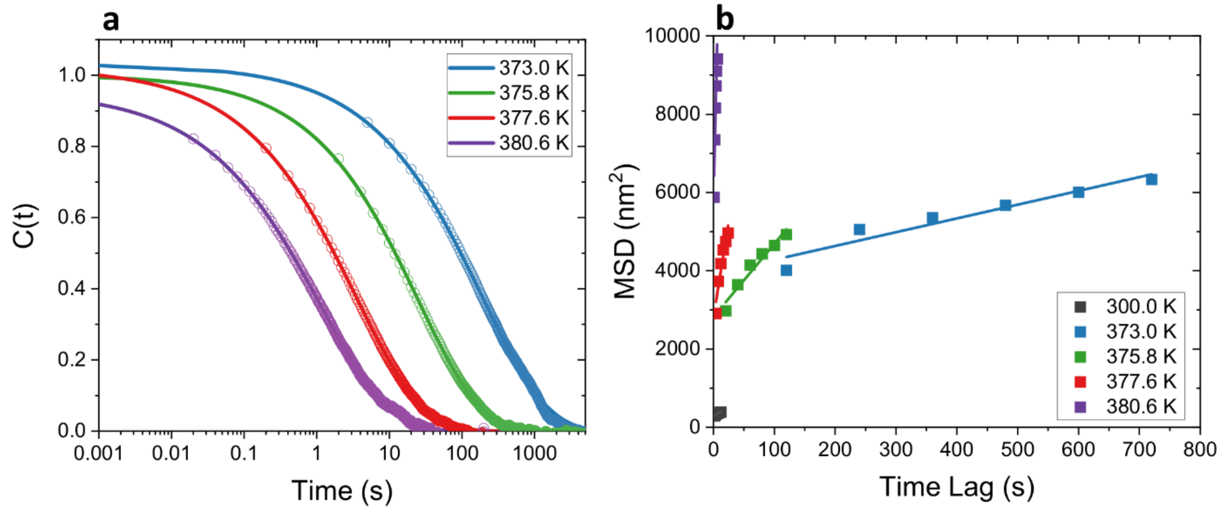

Supplementary Fig. 4. (a) Quasi-ensemble (QE) ACFs obtained from averaging all single molecule linear dichroism ACFs. The QE ACFs were fit to the same stretched exponential equations as described in Methods to obtain $\tau_{fit,QE}$, β_{QE}, and $\tau_{c,QE}$. β_{QE} values are lower than single molecule median values, ranging from 0.46-0.50 rather than 0.61-0.66 but $\tau_{c,QE}$ values are very similar to median τ_c values obtained from single molecules. (b) Quasi-ensemble MSDs obtained from averaging all single molecule MSDs obtained at TBF = τ_{fit} for each temperature. Lines are best fits to the first six points in the MSD, which are used to obtain $D_{T,QE}$ values. $D_{T,QE}$ values are larger than single molecule median D_T values, as shown in **Supplementary Fig. 6**.

Supplementary 5 - Imaging Fluorescence Correlation Microscopy Data

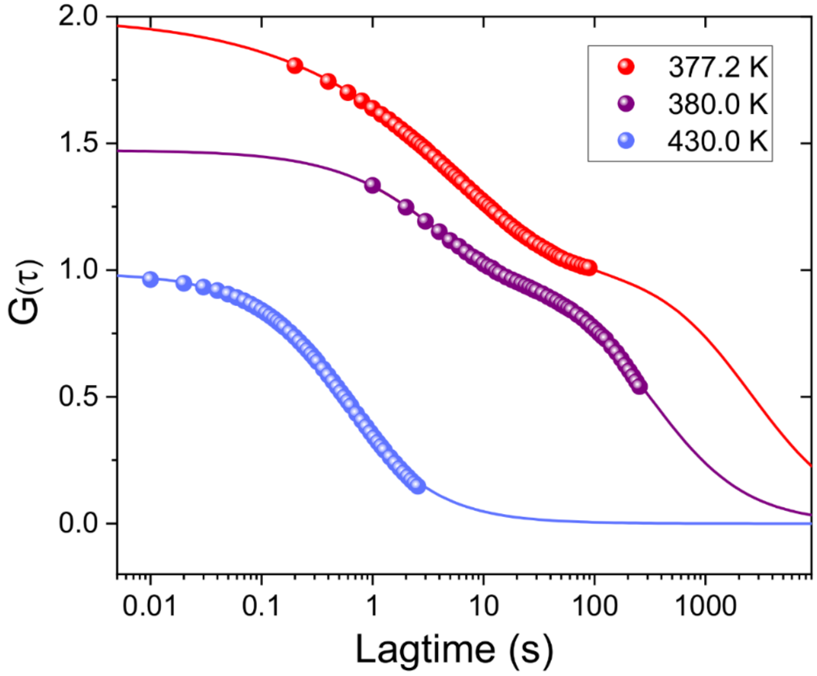

Supplementary Fig. 5. Typical imFCM data obtained for pPDI in 168 kg/mol polystyrene and fits to Equation 2 in the Main Text. At intermediate temperatures, such as 380.0 K, both modes of the relaxation can be captured simultaneously with a movie of practical length ($\approx$ 5000-10000 frames). At other temperatures, such as 430.0 and 377.2 K, only a single mode (translation and rotation, respectively) is accessible for movies of similar length. To conveniently acquire data leading to accurate values in both modes, frame rates were adjusted to optimize data collection for a given mode, as shown in the 430.0 and 377.2K data above. For the purposes of this work, all rotational and translational imFCM measurements were recorded and fit separately.

Supplementary 6 – Additional Debye-Stokes-Einstein Breakdown Information

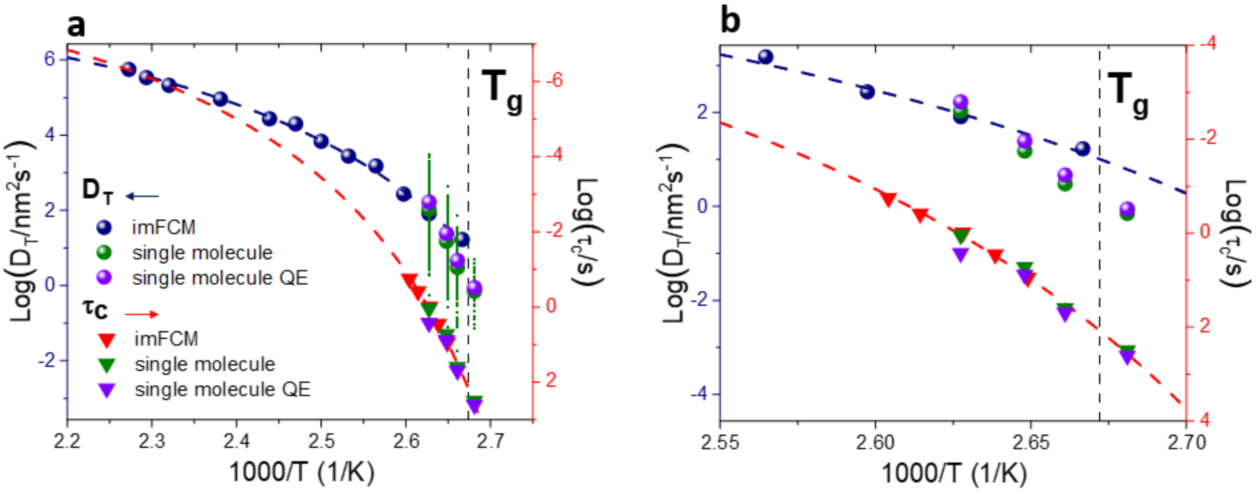

Supplementary Fig. 6. (a) The same data presented in Fig. 1c in the main text with the full distribution of diffusion coefficients from single molecule measurements shown as small green symbols around the median value (large green circles) as well as results from quasi-ensemble analysis of data shown in Supplementary Fig. 4 (purple circles and triangles). (b) The same data shown in (a), excluding full distributions, shown over the temperature range interrogated with single molecule measurements.

Supplementary 7

Supplementary Note: Debye-Stokes-Einstein Behavior and Assumptions

The DSE and SE equations are given by $\tau_c = \frac{4\pi\eta r_s^3}{3kT}$ and $D_T = \frac{kT}{6\pi\eta r_s}$, respectively, where τ_c and D_T are the rotational correlation time and translational diffusion coefficient, respectively, T is temperature, r_s is hydrodynamic radius of the probe, and η is host viscosity. Using the assumptions inherent in these equations describing the motion of a sphere in a hydrodynamic continuum, $D_T\tau_c = \frac{2}{9}r_s^2$.[9–11] Translational data obtained from imFCM and Volgel-Fulcher-Tamman extrapolation of rotational data converge at the three highest temperatures probed to a value $D_T\tau_c \approx 0.25$ nm^2, yielding a physically reasonable pPDI radius estimate of 1.06 nm in 168 kg/mol polystyrene.

With this in mind, together with findings described in **Supplementary 3**, **Supplementary Fig. 7** shows $D_T\tau_c$ vs TBF in terms of (a) τ_{fit} and (b) real time. Plotting this value vs. TBF in terms of τ_{fit} leads to data that exhibits a degree of collapse, with the same functional form as the data in **Supplementary Fig. 3b**. When interrogated, on average, over a timescale associated with ≈ 30 τ_{fit} (when TBF/τ_{fit} ≈ 1), independent of temperature, molecules explore a region of ≈ 100 nm^2, an area associated with rotational-translational decoupling of 2.6 orders of magnitude when compared to the expected value of 0.25 nm^2. For the two lowest temperatures explored, the value of $D_T\tau_c$ shows signs of plateauing with increasing TBF, perhaps suggesting the onset of distinct behavior at these low temperatures. At the two higher temperatures, no sign of a turnover is present, and the data can be extrapolated to the point at which no breakdown would be present, a value of TBF ≈ 150 τ_{fit}. This value is in relatively close agreement with extrapolations from solely rotational measurements performed on pPDI in 168 kg/mol polystyrene at similar temperatures, which suggested full ergodicity restoration at ≈ 80 τ_{fit}.[2]

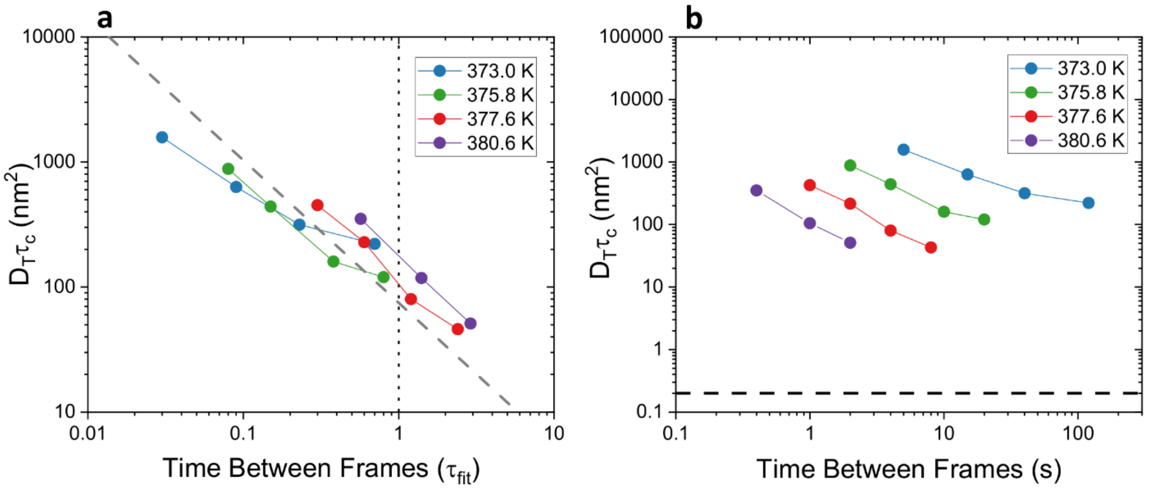

Supplementary Fig. 7. (a) $D_T\tau_c$ vs. TBF in terms of τ_{fit} at each temperature. Gray dashed line is the same line shown in Supplementary Fig. 3b. Gray dotted line emphasizes results at TBF/τ_{fit} ≈ 1. (b) $D_T\tau_c$ vs. TBF in terms of seconds. Black dashed line represents $D_T\tau_c$ value if no DSE breakdown were present.

Supplementary 8

Supplementary Note: Additional Single Molecule Scatter Plots

As discussed in the Main Text, single molecule data at each measured temperature showed limited correlation between rotational dynamics as reflected in τ_c and translational dynamics as reflected in D_T. To assess to what extent this may be related to the fact that translational dynamics were typically assessed for only a subset of the full time over which rotations were assessed, several approaches were taken. First, instead of comparing τ_c and D_T, τ_{fit} and D_T were compared (**Supplementary Fig. 8a**). Because τ_{fit} describes the initial decay of the linear dichroism (LD) autocorrelation function (ACF), it does not include contributions from the long-time tail often present in the LD ACFs. Despite this, correlation was similar between both approaches (Pearson's R = 0.0035 for τ_c vs. D_T and R = -0.056 for τ_{fit} vs. D_T for data collected at 375.8 K, for example). Additionally, as R_g requires no fitting in the way that D_T does, τ_c and R_g were compared to determine rotational and translational correlation outside of the context of MSD fits (**Supplementary Fig. 8b**). Correlation is similarly low for τ_c and D_T as for τ_c and R_g (Pearson's R = -0.17 for τ_c vs R_g at 375.8 K). Further, to attempt to remove the role of trajectory lengths that differed in rotation and translation, we explicitly only considered frames over which D_T was interrogated; this data also revealed limited correlation (**Supplementary Fig. 8c**). Similarly, a per-molecule approach wherein only the exact frames over which molecules were tracked for translational analysis were used to determine rotational correlation times (**Supplementary Fig. 8d**). All reveal similar scatter plots and limited correlation, indicating that differing trajectory lengths are not the primary driver of this lack of correlation seen also in **Fig. 2** in the Main Text. Finally, results obtained from molecules identified through the frame rate sweep (**Supplementary 3**) were analyzed in terms of τ_{fit} vs. D and τ_c vs. D (**Supplementary Fig. 8e**) as well as via $D_T\tau_c$ vs. D_T (**Supplementary Fig. 8f**). Somewhat stronger correlations were found via frame rate sweep compared to molecules assessed through the standard approach using TBF = τ_{fit}; for example, at 375.8 K Pearson's R = 0.0035 from τ_c vs. D_T at TBF = τ_{fit}, and R = - 0.17 for the comparable frame rate sweep. For $D_T\tau_c$ vs D_T, Pearson's R = 0.72 for TBF = τ_{fit}, and R = 0.86 for the comparable frame rate sweep.

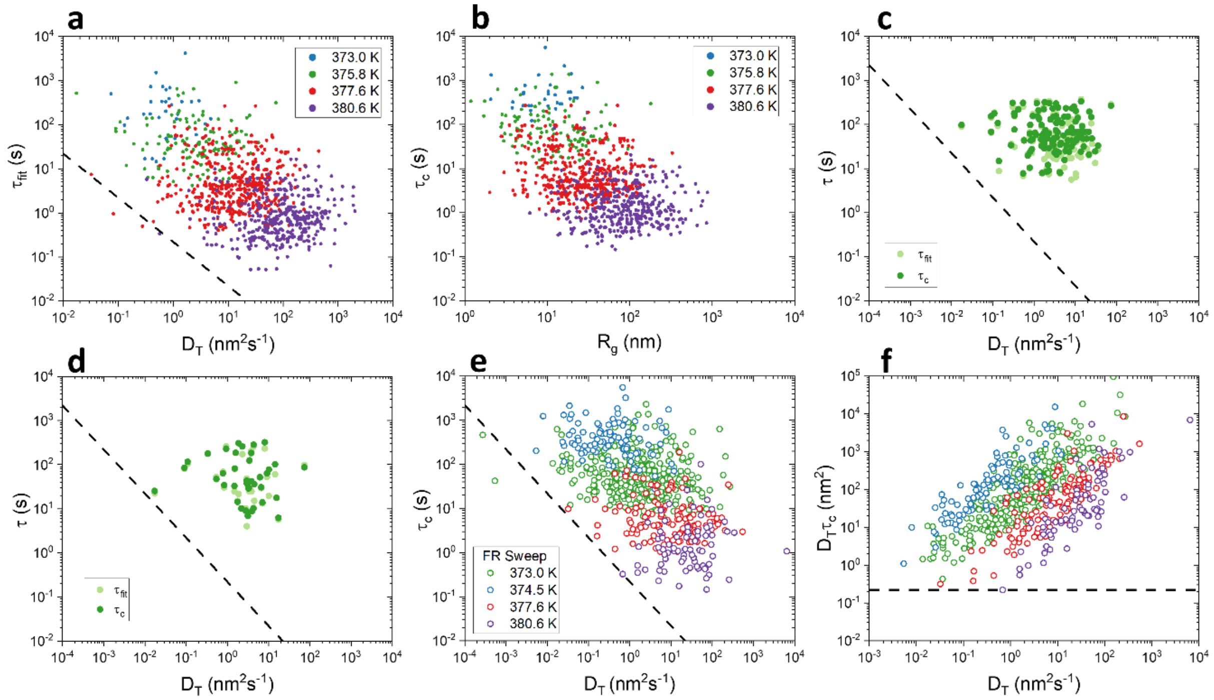

Supplementary Fig. 8. (a) τ_{fit} vs. D_T single molecule scatter plots at each measured temperature. Dashed black line represents the DSE expectation for all graphs where such a line is present. The trend is the same as for the τ_c vs. D_T scatter plot shown in the Main Text in Fig. 2a. (b) τ_c vs. R_g single molecule scatter plot. The trend is similar to that seen in τ_c vs D_T. (c) Scatter plot of τ_{fit} and τ_c vs. D_T for data collected at 375.8 K when molecules are analyzed rotationally only over the frames used to track translation, reducing the total number of frames analyzed in rotation from 5000 to 1300. Correlation remains similar to that seen in (a) and Fig. 2a in the Main Text. (d) τ_{fit} and τ_c vs. D_T for data collected at 375.8K when molecules were analyzed rotationally only over the exact frames used to track translation per molecule, a reduction in total number of frames analyzed from 5000 to a median of 300 per molecule. Correlation remains similar to that seen in (a) and in Fig. 2a in the Main Text, though fewer molecules can be assessed in this manner as rotational autocorrelation functions cannot always be well fit for such short trajectories. (e) Scatter plots of τ_{fit} and τ_c vs. D_T for molecules identified through frame rate sweep analysis at all temperatures. Correlation remains similar to Fig. 2a in the Main Text. (f) Scatter plots of $D_T\tau_c$ vs. D_T for molecules identified through frame rate sweep analysis at all temperatures. Correlations remain similar to though somewhat stronger than those in the Main Text Fig. 2a and b.

Supplementary 9

Supplementary Note: Translational Diffusion Coefficients of Molecules Stratified by Mobility

As discussed in the Main Text, stratifying molecules based on radius of gyration allows molecules with very slow dynamics to be retained for further analysis. This is in contrast to stratification based on obtained diffusion coefficients from MSD analysis, due to the fact that analysis of such molecules may yield negative, unphysical diffusion coefficients. Below, we show diffusion coefficients, including negative ones, obtained from mobile and immobile molecules, with identification of molecules as mobile or immobile as described in the Main Text. Consistent with expectation, molecules deemed immobile tend to have very small diffusion coefficients with a narrow distribution, and have a much larger relative incidence of negative diffusion coefficients than do mobile molecules (**Supplementary Fig. 9a-d**). When only mobile molecules with positive diffusion coefficients are considered, for those temperatures in which a significant number of molecules are retained (377.6 and 380.6 K), distributions remain similar to those shown in **Fig. 1b** in the Main Text (**Supplementary Fig. 9e**).

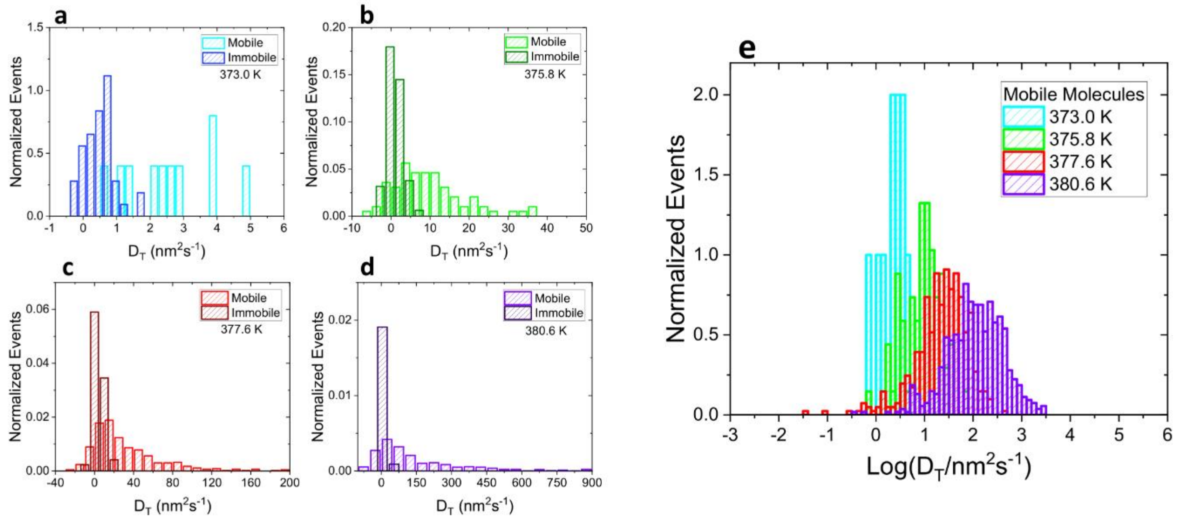

Supplementary Fig. 9. (a-d) Diffusion coefficient distributions of mobile molecules and immobile molecules at (a) 373.0 K, (b) 375.8 K, (c) 377.6 K, and (d) 380.6 K. Molecules that returned a negative diffusion coefficient, indicating large localization error relative to movement are included to illustrate the high proportion of negative D_T molecules flagged as immobile. (e) D_T distributions at each temperature including only molecules identified as mobile.

Supplementary 10 - Additional Characteristics of Mobile and Immobile Molecules

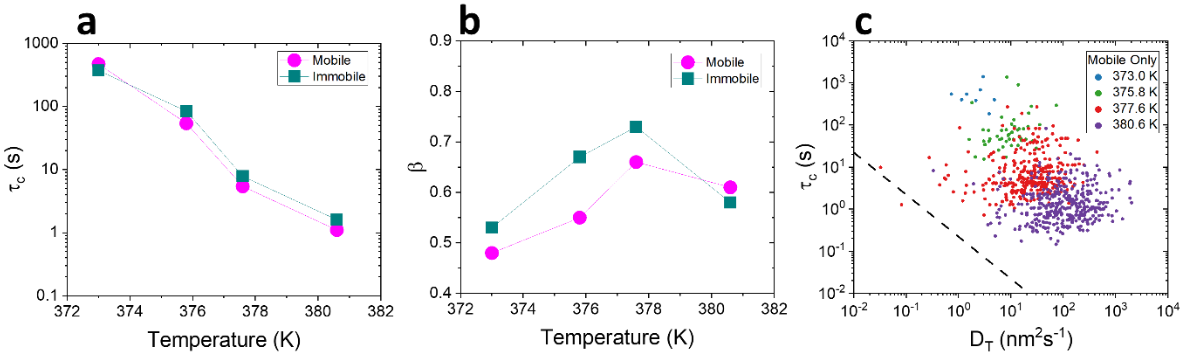

Supplementary Fig. 10. (a) Median τ_c vs. temperature for mobile and immobile molecules. Immobile molecules tend to have larger τ_c values. (b) Stretching exponent β vs. temperature for mobile and immobile molecules. Mobile molecules tend to have lower β values, suggesting these molecules have experienced more dynamic environments, consistent with more translational motion through distinct regions. (c) τ_c vs. D_T single molecule scatter plot analogous to Fig. 2a in the Main Text only for molecules identified as mobile. The trend and correlations are similar to those seen in Fig. 2a of the Main Text, though there are fewer molecules present at the lowest temperatures investigated since many molecules at these temperatures are identified as immobile. Black dashed line indicates DSE/SE expectation.

Supplementary 11 – Step Size Distributions

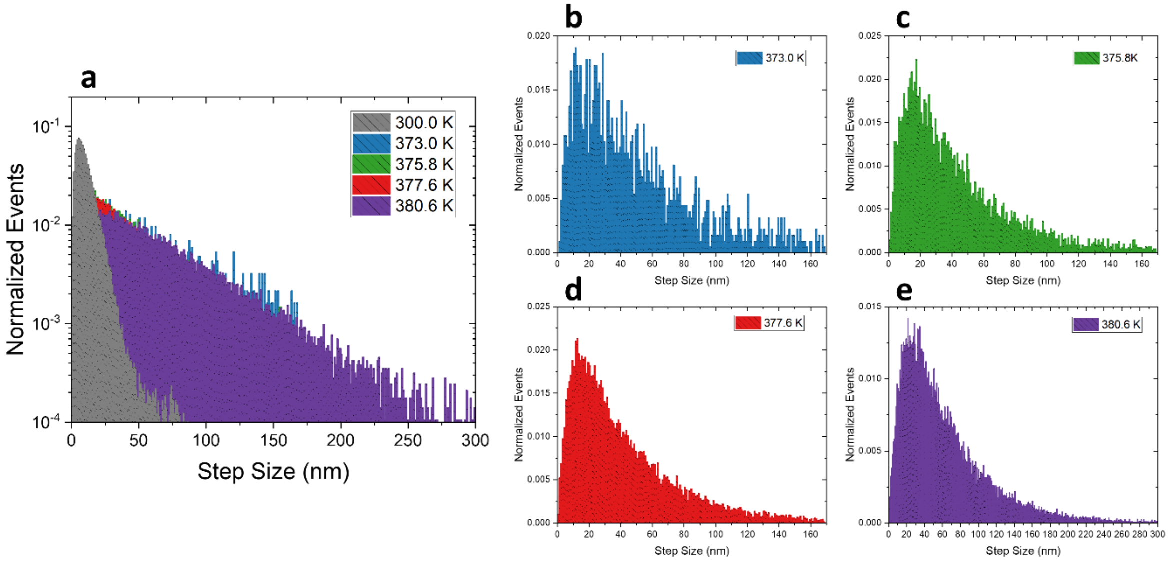

Supplementary Fig. 11. (a) Step size distributions of 300.0 K data (as also shown in Supplementary Fig. 2a) compared to step size distributions of data collected at temperatures near and above T_g at TBF = τ_{fit}. Step size distributions at the higher temperatures exhibit non-Gaussian tails distinct from data collected at 300.0 K. (b-e) Same data presented in (a) with data from each temperature near or above T_g presented separately and on a linear scale.

Supplementary References

1. Paeng, K. & Kaufman, L. J. Single Molecule Experiments Reveal the Dynamic Heterogeneity and Exchange Time Scales of Polystyrene near the Glass Transition. *Macromolecules* **49**, 2876–2885 (2016).
2. Manz, A. S., Paeng, K. & Kaufman, L. J. Single molecule studies reveal temperature independence of lifetime of dynamic heterogeneity in polystyrene. *J. Chem. Phys.* **148**, 204508 (2018).
3. Pennacchietti, F., Gould, T. J. & Hess, S. T. The Role of Probe Photophysics in Localization-Based Superresolution Microscopy. *Biophys. J.* **113**, 2037–2054 (2017).
4. Mortensen, K. I., Churchman, L. S., Spudich, J. A. & Flyvbjerg, H. Optimized localization analysis for single-molecule tracking and super-resolution microscopy. *Nat. Methods* **7**, 377–381 (2010).
5. Andor. How Andor iXon EMCCD Cameras Present Data. https://andor.oxinst.com/learning/view/article/count-convert
6. Michalet, X. Mean square displacement analysis of single-particle trajectories with localization error: Brownian motion in an isotropic medium. *Phys. Rev. E* **82**, 04194 (2010).
7. Flier, B. M. I. *et al.* Heterogeneous diffusion in thin polymer films as observed by high-temperature single-molecule fluorescence microscopy. *J. Am. Chem. Soc.* **134**, 480–488 (2012).
8. Paeng, K., Park, H., Hoang, D. T. & Kaufman, L. J. Ideal probe single-molecule experiments reveal the intrinsic dynamic heterogeneity of a supercooled liquid. *Proc. Natl. Acad. Sci. U. S. A.* **112**, 4952–4957 (2015).
9. Einstein, A. On the Movement of Small Particles Suspended in Liquids at Rest Required by the Molecular-Kinetic Theory of Heat. *Ann. Phys.* **17**, 549–560 (1905).
10. Debye, P. J. W. *Polar Molecules.* The Chemical Catalog Company, New York, NY (1929).
11. Cicerone, M. T. & Ediger, M. D. Enhanced translation of probe molecules in supercooled o-terphenyl: Signature of spatially heterogeneous dynamics? *J. Chem. Phys.* **104**, 7210–7218 (1996).
